# MicroRNAs-Mediated Regulation of Skeletal Muscle GLUT4 Expression and Translocation in Insulin Resistance

**DOI:** 10.1155/2017/7267910

**Published:** 2017-03-27

**Authors:** João Victor Esteves, Francisco Javier Enguita, Ubiratan Fabres Machado

**Affiliations:** ^1^Department of Physiology and Biophysics, Institute of Biomedical Sciences, University of São Paulo, São Paulo, SP, Brazil; ^2^Instituto de Medicina Molecular, Faculdade de Medicina, Universidade de Lisboa, 1649-028 Lisboa, Portugal

## Abstract

The solute carrier family 2 facilitated glucose transporter member 4 (GLUT4) plays a key role in the insulin-induced glucose uptake by muscle and adipose tissues. In prediabetes and diabetes, GLUT4 expression/translocation has been detected as reduced, participating in mechanisms that impair glycemic control. Recently, a class of short endogenous noncoding RNAs named microRNAs (miRNAs) has been increasingly described as involved in the posttranscriptional epigenetic regulation of gene expression. The present review focuses on miRNAs potentially involved in the expression of GLUT4 expression, and proteins related to GLUT4 and translocation in skeletal muscle, seeking to correlate them with insulin resistance and diabetes. So far, miR-21a-5p, miR-29a-3p, miR-29c-3p, miR-93-5p, miR-106b-5p, miR-133a-3p, miR-133b-3p, miR-222-3p, and miR-223-3p have been reported to directly and/or indirectly regulate the GLUT4 expression; and their expression is altered under diabetes-related conditions. Besides, some miRNAs that have been linked to the expression of proteins involved in GLUT4 translocation machinery in muscle could also impact glucose uptake. That makes these miRNAs promising targets for preventive and/or therapeutic approaches, which could improve glycemic control, thus deserving future new investigations.

## 1. Introduction

Diabetes mellitus (DM) is a metabolic disorder with high prevalence in the world [1]. It is characterized by hyperglycemia resulting from defects in insulin secretion and/or action. The most prevalent forms of the disease are type 1 diabetes mellitus (T1DM) and type 2 diabetes mellitus (T2DM). T1DM results primarily from the lack of insulin secretion due to autoimmune destruction of pancreatic *β* cells, whereas T2DM primarily results from hormone resistance in target tissues, particularly in liver, adipose tissue, and skeletal muscle, which can lead to impaired insulin secretion [2].

Several studies have sought to clarify the main molecular mechanisms involved in the pathophysiology of T2DM. It is known that insulin resistance is important to the development and maintenance of glycemic homeostasis loss [3], a phenomenon in which skeletal muscle plays a key role [4]. The skeletal muscle is the main tissue responsible for insulin-stimulated glucose disposal and the major site of peripheral insulin resistance [4]. In muscles, as in the adipose tissue, the insulin-stimulated glucose uptake is performed through the solute carrier family 2, facilitated glucose transporter member 4 (GLUT4), which is rapidly translocated to the plasma membrane in response to the hormone [5, 6]. Although the skeletal muscle glucose disposal can be reduced by impaired GLUT4 translocation [6, 7], long term established reduction in the glucose uptake has currently been related to a defective glucose transporter gene and/or protein expression [8].

Recently, a new element in the pathophysiology of diabetes emerged, receiving great attention. It is a class of small endogenous noncoding RNAs termed microRNAs. MicroRNAs (miRNAs) are regulatory small noncoding RNAs (~22 nucleotides) that bind to the 3′UTR region of mRNAs, destabilizing them or inhibiting their translation, and thus working as negative posttranscriptional regulators of gene expression [9]. Hence, miRNAs participate as important regulators of biological processes, including cellular differentiation, proliferation, apoptosis, and metabolism, as well as tissue development [9]. Thereafter, alterations in the expression of miRNAs have been observed in numerous cellular dysfunctions, and many studies have been performed to investigate the role of miRNAs in the pathophysiology of diabetes [10–12].

This review summarizes the potential participation of miRNAs in the regulation of GLUT4 protein (codified by the* SLC2A4* gene) and proteins related to its translocation, in skeletal muscle, exploring their involvement in the pathophysiology of diabetes, as well as their potential role in preventive or therapeutic approaches for diabetes.

## 2. Skeletal Muscle, GLUT4, and Glycemic Homeostasis

Skeletal muscle makes up approximately 40% of the total body mass. The main function of skeletal muscle is contraction-related, for which the main energetic substrate is glucose. Skeletal muscle is a great consumer of extracellular glucose, which increases under insulin- and contraction-stimulation. In healthy subjects, skeletal muscle accounts for up to ~80% of glucose disposal under insulin-stimulated conditions, as it occurs in postprandial state, thus displaying a fundamental role in glycemic homeostasis [3].

The glucose transport in mammalian tissues occurs primarily by facilitated diffusion, a process that needs carrier proteins. In the early eighties, it was demonstrated that insulin was able to increase glucose transport in adipocytes [13] and skeletal muscle [14]. Later on, an insulin-regulatable glucose transporter was identified in these tissues [15], and a cDNA encoding this glucose transporter was obtained in rat, mouse, and human [16–20]. This insulin-responsive glucose transporter was ultimately named GLUT4, based on the chronological order in which the various isoforms of GLUTs were characterized.

The GLUT4 protein (codified by the gene* solute carrier family 2 member 4*,* SLC2A4*) is the major glucose transporter of brown and white adipose tissues and of skeletal and cardiac muscles [21]. Under basal conditions, that is, without insulin stimulus, a great amount of GLUT4 is restrained in tubule-vesicular structures named GLUT4 storage vesicles (GSVs) [22]. Under insulin stimulus, the GSVs are translocated to the cell surface, rapidly increasing the GLUT4 density at plasma membrane, and, consequently, the glucose uptake [5, 6, 23]. Insulin signaling regulates the several steps of GLUT4 translocation, including GSVs releasing from intracellular retention, trafficking, tethering, and, finally, docking and fusion into the cell membrane. In brief, insulin binds to its receptor, activating intrinsic kinase activity, leading to tyrosine phosphorylation of insulin receptor substrate 1 (IRS1) (the predominant IRS isoform in skeletal muscle) and activating phosphatidylinositide 3-kinase (PI3K). PI3K signaling bifurcates into the Rac1 (a Rho GTPase) and the PDK1 (phosphoinositide-dependent protein kinase) pathways, which converge to GLUT4 translocation, both of which ultimately stimulate small GTPases [6, 24, 25].

Rac1 pathway involves rearrangement of actin-filaments and cortical actin branching, mediated by actin-related proteins 2/3 (ARP2/3) complexes [26], which provides a path along which GSVs are transported, and is crucial for retention and fusion of GSV beneath the plasma membrane [27]. PDK1 pathway leads to activation of AKT (particularly isoform AKT2) and of the atypical protein kinases C zeta and lambda (PKC*ζ*/*λ*) [28], all involved in the GSV translocation. AKT-mediated signal involves downstream participation of the RAB-GTPase-activating proteins named TBC1D4 (also known as AS160) and TBC1D1. Several RAB-GTPases are targets for TBC1D4 in muscle cells [29–33] and are involved in intracellular vesicle traffic through engagement of mechanical effectors responsible for vesicle budding, mobilization, and fusion [34]. Finally, unconventional myosin-Ic (MYO1C) and SNAP-associated receptor (SNARE) proteins such as vesicle-associated membrane protein 2 (VAMP2), syntaxin-4 (STX4), synaptosomal-associated protein 23 (SNAP23), syntaxin-binding protein 3 (STXBP3), and syntaxin-binding protein 4 (STXBP4) were described as essential elements for insulin-mediated tethering and fusion of GSVs in muscle cells [6, 23, 35, 36].

Besides, in skeletal muscle, contraction-induced GLUT4 translocation also occurs, involving at least two proteins: AMP-activated protein kinase (AMPK) and Ca^2+^/calmodulin-dependent protein kinase (CaMK2) [37], which can operate in addition to insulin-triggered ones. Thus, GLUT4 is the major mediator of extracellular glucose clearance, playing a key role in the regulation of glycemic homeostasis [3, 6]. In addition to the acute effect of insulin and muscle contraction upon GLUT4 translocation, it has also been demonstrated that both stimuli are able to increase* Slc2a4*/GLUT4 expression, by, respectively, activating or inhibiting enhancer or repressor transcriptional factors [38–40].

Considering what was described above, it becomes evident that insulin and muscle contraction modulate glycemic homeostasis by regulating both translocation and expression of GLUT4.

## 3. Skeletal Muscle GLUT4 Expression and Translocation: From Insulin Resistance to T2DM

In prediabetes and T2DM, reduced insulin-stimulated glucose uptake by skeletal muscle is a current feature [3, 41]. Additionally, in T1DM, skeletal muscle glucose disposal can also be reduced because of glucotoxicity and/or hyperinsulinemia [42], pointing out that insulin therapy is obligatory. Thus, under all conditions related to impaired glycemic control, insulin resistance is involved, and, thus, altered skeletal muscle GLUT4 expression and translocation must be involved.

Since the characterization of GLUT4, and considering its central role in glycemic homeostasis, several studies have been performed attempting to understand the role of the skeletal muscle* SLC2A4*/GLUT4 expression and translocation in the pathophysiology of insulin resistance and/or diabetes. However, the interpretation of the results must be carefully performed, because muscle tissues show multiple fiber types, displaying different rates of insulin sensitivity and GLUT4 expression. Red (type I, slow-twitch, oxidative) fibers are significantly more insulin-responsive than white (type IIa/b, fast-twitch oxidative/glycolytic) muscle fibers and have higher levels of GLUT4 protein [43, 44]. So, the skeletal muscle participation upon glycemic homeostasis must take the whole-body proportion between white and red muscles into account.

Skeletal muscle* SLC2A4*/GLUT4 expression under conditions of impaired glycemic homeostasis has been investigated since the early nineties. In prediabetes, T2DM and T1DM experimental models, GLUT4 expression in skeletal muscle has been regularly detected to be reduced [42, 45–49]. Besides, transgenic animals with muscle-specific inactivation of GLUT4 show a diabetic phenotype [50, 51], and the muscle-specific GLUT4 overexpression in diabetic animals improves glycemic control [52, 53], pointing out the fundamental role of GLUT4 in glycemic homeostasis [7]. In humans, although some pioneering studies reported that* SLC2A4*/GLUT4 expression was unchanged in skeletal muscles of prediabetes and T2DM subjects [54, 55], other studies reported a reduction of GLUT4 [56], which was further confirmed in more sensitive quantitative analyses of GLUT4 [57, 58]. Therefore, repressed* Slc2a4*/GLUT4 expression in skeletal muscle becomes a focus in the pathophysiology and treatment of impaired glycemic homeostasis conditions [8, 59–61]. Finally, in insulin-deficient animals [42, 62] and T1DM humans [63, 64] under insulin therapy, the* Slc2a4*/GLUT4 expression seems to be unaltered, as compared to nondiabetic state, reinforcing the important role of the glycemic homeostasis in the regulation of this gene.

Insulin signaling damage also plays a role in insulin resistance of skeletal muscle, by impairing GLUT4 storage vesicles (GSV) translocation. In T2DM humans, reduced activation of early steps of insulin signaling, such as IRS1/PI3K [24, 28], and downstream steps such as activation AKT, PKC-zeta, and TBC1D4 [28, 65, 66] have been observed to be impaired, thus compromising insulin-stimulated GSV translocation. On the other hand, in T2DM, muscle contraction-stimulated glucose transport is preserved, and the activity of proteins related to contraction-induced GSV translocation, such as AMPK and CAMKII, has been described as unaltered [37]. Importantly, despite reduced activation of several proteins related to the GSV translocation machinery, the expression (cellular content) of these proteins is unaltered in T2DM, except by the PKC-zeta that was found reduced [28].

Behavioral (diet and exercise) and pharmacological (metformin, thiazolidinediones, glimepiride, insulin, resveratrol, and quercetin) interventions that improve glycemic control have been described as able to increase* Slc2a4*/GLUT4 expression and translocation [8, 39, 42, 49, 67–72]. Besides, although some insulin sensitizers were developed without the knowledge of their effects on the machinery of the GSV translocation [24], some of them such as exercise, metformin, and thiazolidinediones have revealed improvement of some steps of the insulin signaling cascade [24, 28].

In both T1DM and T2DM, muscle glucose disposal is variably impaired, and, because of that, skeletal muscle was emphasized by Coleman et al. as an important target for delaying diabetic complications [73]. The present data highlight the importance of increasing muscle* SLC2A4* gene expression to improve glycemic control, since the GLUT4 density in the GSV is primordial for an effective GLUT4 translocation. In fact,* Slc2a4* gene becomes a promising target for pharmacogenomics of insulin resistance [8], and, for that, it is mandatory to unravel mechanisms related to the* SLC2A4* gene expression, such as its epigenetic regulation by microRNAs.

## 4. MicroRNAs (miRNAs)

MicroRNAs (miRNAs) are a class of short (~22 nt) endogenous noncoding RNAs that participate in the epigenetic regulation of gene expression [9]. Since the discovery of the first miRNA (named lin-4) in nematode* Caenorhabditis elegans* [74, 75] and the identification of the first miRNA in humans (named let-7) [76], hundreds of miRNAs have been found in both animals and plants and have revolutionized the understanding of the regulation of gene expression.

The vast majority of miRNAs are initially transcribed by RNA polymerase II resulting in a long primary transcript called pri-miRNA. The pri-miRNA is subsequently processed: firstly in the nucleus, by the Drosha–DGCR8 complex, to a ~60–70 nt precursor miRNA (pre-miRNA) hairpin-loop structure, which is exported to the cytoplasm where the Dicer–TRBP complex will generate a ~22 nt double-stranded miRNA [77]. One strand of this duplex, representing a mature miRNA, is then incorporated into the miRNA-induced silencing complex (miRISC). As part of miRISC, miRNAs imperfectly base-pair with sequences in the 3′ untranslated region (UTR) of target mRNAs and inhibit protein synthesis by either repressing translation or promoting mRNA deadenylation and decay [77]. This interaction is nucleated by perfect Watson-Crick base pairing of nucleotides 2–7 at the 5′ end of the miRNA (termed “seed sequence”) with a complementary seed match site in the 3′-UTR of the target mRNA [78]. However, it is important to note that the base pairing may not occur only in the 3′-UTR, but also in the open reading frame (ORF) or 5′-UTR of the mRNA target [79]. Importantly, a single miRNA can regulate the expression of hundreds of genes, and the expression of a single gene can be regulated by multiple miRNAs [80]. Moreover, computational predictions suggest that more than 60% of all mammalian protein-coding genes may be regulated my miRNAs [81]. Indeed, miRNAs have been implicated in the regulation of many key biological processes, and, miRNA deregulation is a common feature in several pathophysiological conditions, including metabolic disorders such as diabetes [11, 12, 82–84], suggesting that miRNAs could serve as targets for preventive or therapeutic interventions.

## 5. miRNAs and Skeletal Muscle

Many miRNAs are expressed in a tissue-specific manner. This concept was confirmed in a study by Lagos-Quintana et al. [85] showing that miR-1, miR-122-5p, and miR-124-3p expression was restricted to striated muscle, liver, and brain, respectively. Currently, a tissue-specific miRNA is defined as a miRNA that is expressed in a specific tissue at a level that is at least 20-fold higher than it is expressed in all other tissues [86]. Some miRNAs, including miR-1, miR-133a/b-3p, and miR-206-3p, are highly expressed in skeletal muscle, corresponding to nearly 25% of total miRNA expression in skeletal muscle, and, thus, they are habitually referred to as muscle-specific miRNAs or myomiRs [87]. Other myomiRs described include miR-208a-3p, miR-208b-3p, and miR-499-5p [88]. Most myomiR family members are expressed in both the heart and the skeletal muscle, except for miR-208a-3p, which is cardiac-specific [88], and miR-206 which is only found in skeletal muscle, mainly in slow-twitch muscles such as the soleus [89]. In addition, miR-208b-3p and miR-499-5p, encoded by myosin genes, may be useful markers of the muscle fiber type [88].

Several of these miRNAs are organized under bicistronic clusters on the same chromosome (i.e., miR-1-1/133a-2, miR-1-2/133a-1, and miR-206/133b) and are transcribed together [90]. These myomiRs are controlled by fundamental myogenic regulatory factors, including myoblast determination protein 1 (MYOD1), myogenic factor 5 (MYF5), myogenic factor 6 (MYF6), and myogenin (MYOG), as well as serum response factor (SRF) and myocyte enhancer factor 2 A/B/C/D (MEF2A/B/C/D) [91, 92]. Together, they regulate the key processes of myogenesis, including myoblast/satellite cell proliferation and differentiation [91]. Furthermore, myomiRs are modulated during multiple biological processes involved in skeletal muscle growth, development, and maintenance, including hypertrophy and atrophy [87, 93].

Identifying the role and regulation of skeletal muscle miRNAs expression during several phases of muscle development, as well as under health impaired conditions, will significantly enhance the understanding of skeletal muscle biology and may result in new therapies to target muscle diseases or chronic diseases associated with impaired muscle function [94].

## 6. miRNAs and Proteins of the GLUT4 Translocation Machinery in Insulin Resistance

Over the last few years, several known miRNAs were described as involved in the pathophysiology of diabetes [11, 95], especially in the impaired glycemic homeostasis [10, 80, 96]. Some studies related changes in miRNAs expression in skeletal muscle of diabetic Goto-Kakizaki rats (decreased miR-10b-5p, miR-24-3p) and T2D humans (decreased miR-133a-3p and miR-206), but they did not relate these miRNAs to any target mRNA [97–99]. In a step by step review of the protein related to the GLUT4 translocation machinery in skeletal muscle, several miRNAs have been potentially correlated to some target genes.

Regarding the early steps of insulin signaling, the* INSR* mRNA was suggested to be target for miR-let-7f-5p in T2DM mice [100] and for miR-15b-5p in T2DM humans [101]. Besides, it was reported that* INSR* gene expression was downregulated in miR-135a-5p transfected C2C12 cells [102].* IRS1* and/or* IRS2* mRNAs were correlated with (1) let-7f-5p in T2DM mice [100]; (2) miR-15b-5p in T2DM humans [101]; (3) miR-29a-3p in IR-obese mice [103]; (4) miR-135a-5p in T2DM mice [104]; and (5) miR-144-3p in T2DM rats [105].

*PI3KR1* mRNA was proposed to be regulated only by miR-15b-5p in T2DM humans [101].* AKT2* mRNA reduction was correlated to reduced expression of miR-23a and miR-107 after loss of weight in obese dogs [106], suggesting an unexpected and incomprehensible increasing of AKT2 in the obese insulin resistant dogs before the weight loss. Besides, miR-29/a/b/c-3p regulation of* AKT2* was also suggested in GK rats [107]. Finally, regulation of some proteins downstream the PI3K has also been related to miRNAs: (1)* MTOR* and* P70S6K1* were proposed to be regulated by reduction of miR-16-5p in obese rats and mice [108]; (2)* MAPK14* expression was related to miR-24-3p in GK rats [99]; and (3)* RAC1* decrease was correlated with miR-23a and miR-107 decreasing after weight loss of obese dogs [106]. Additionally, phosphorylation of AKT and TBC1D4 was related to decreased miR-194-5p [109] and increased miR-494-3p [110], respectively; however, this effect cannot be attributed to a direct miRNA epigenetic regulation. Finally, there is no report relating miRNAs and posttranscriptional regulation of* AMPK* and* CAMK2* genes in skeletal muscle of insulin resistant animals or humans.

Table 1 summarizes the miRNAs potentially involved in the regulation of proteins that are related to GLUT4 translocation machinery in skeletal muscle tissue/cells under insulin resistant conditions.

Finally, it is important to highlight that most epigenetic regulation described here were not validated, but just reported as correlated regulation. So, future studies must be conducted to validate that miRNAs participate in the regulation of proteins of the GLUT4 translocation machinery in the insulin resistance. It is also important to emphasize that these recent studies on miRNA regulation of proteins of the insulin signaling cascade have described alterations in mRNA/protein content in insulin resistant conditions, which do not match the paradigm that insulin resistance alters only the activation of these proteins, but not their content [28].

## 7. miRNAs Related to the* SLC2A4*/GLUT4 Expression in Insulin Resistance

To our knowledge, under conditions related to insulin resistance and/or diabetes, just a few studies have investigated the regulation of GLUT4 expression by miRNAs. These studies were not performed uniquely in skeletal muscle, but some of them were conducted in adipose and cardiac tissues or cells, and we are including them in the present analysis. The GLUT4 regulation by miRNAs include (1) confirmed direct effect upon* Slc2a4* mRNA expression; (2) confirmed direct effect upon a target that regulates* Slc2a4* mRNA expression; and (3) predictable direct effect by in silico analysis and correlation between expression of GLUT4 and the miRNA.

Confirmed direct modulations of* Slc2a4*/GLUT4 expression were demonstrated for miR-93-5p, miR-223-3p, and miR-106b-5p [111–114].

An elegant study has shown that miR-93-5p was upregulated in subcutaneous adipose tissue from polycystic ovary syndrome (PCOS) and non-PCOS insulin resistant women, and that miR-93-5p expression correlated negatively with GLUT4 and positively with HOMA-IR in these women [111]. Besides, overexpression of miR-93-5p in both human and 3T3-L1 adipocytes decreased GLUT4 protein expression; and, conversely, the inhibition of miR-93-5p increased GLUT4 expression [111]. Besides, by means of luciferase assay, this study confirmed the direct regulation of* Slc2a4* by miR-93-5p [111].

Another miRNA confirmed as a direct regulator of* Slc2a4*/GLUT4 is the miR-223-3p, which has been described as upregulated in adipose tissue of women with insulin resistance [112]. In fact, overexpression of miR-223-3p in human adipocytes was reported to inhibit insulin-stimulated glucose uptake and to decrease GLUT4 protein content [112]. Similarly, upregulation of miR-223-3p was observed in heart of T2DM patients [113]. However, the overexpression of miR-223-3p in cardiomyocyte from neonatal rats was accompanied by increased GLUT4 expression [113], revealing a paradoxical effect as compared to that observed in adipocyte [112].

Recently, a direct negative effect of miR-106b-5p upon* Slc2a4* was demonstrated by luciferase assay, in muscle L6 cells. In these cells, overexpression of miR-106b-5p downregulated the GLUT4 content and decreased the glucose consumption and uptake; and, conversely, knockdown of miR-106b-5p increased the levels of GLUT4 and glucose consumption in this cell [114].

Indirect regulation of* Slc2a4* mRNA expression may be related to miR-222-3p, miR-133a/b-3p, miR-21a-5p, and miR-29a-3p [115–118].

In omental adipose tissue from women with gestational diabetes mellitus miR-222-3p expression was found upregulated; besides that, miR-222-3p expression was negatively correlated with estrogen receptor 1 (ESR1) and GLUT4 contents and positively correlated with serum estradiol levels [115]. In addition,* Esr1* mRNA was shown to be a target for miR-222-3p in 3T3-L1 cells, in which estradiol increased miR-222-3p expression and reduced ESR1 and GLUT4 proteins [115]. Besides, miR-222-3p silencing in 3T3-L1 adipocytes was seen to increase ESR1 and GLUT4 expression, as well as the insulin-stimulated glucose uptake [115]. Regarding that, our group demonstrated that ESR1 is a potent enhancer of* Slc2a4*/GLUT4 expression in 3T3-L1 adipocytes [119], supporting the proposal of an ESR1-mediated effect of miR-222-3p upon GLUT4.

Regulation of GLUT4 expression has also been related to the typical myomiRs miR-133a-3p and miR-133b-3p, which were reported to act indirectly upon* Slc2a4*/GLUT4 expression. In fact, miR-133 was observed to target the* Klf15* mRNA (Krüeppel-like factor 15), inhibiting this transcriptional factor, which is an enhancer of* Slc2a4* expression; thus miR-133 leads to the reduction in GLUT4 expression and in insulin-stimulated glucose uptake in rat cardiomyocytes [116].

Ling and colleagues have suggested that miR-21a-5p targets the* Pten* mRNA (phosphatase tensin homologue), since overexpression of miR-21a-5p in 3T3-L1 adipocytes decreases PTEN protein, despite unchanged* Pten* mRNA, and considering that* Pten* 3′UTR has a highly conserved miR-21a-5p recognition element [117]. PTEN is a negative regulator of PI3K/AKT activity [117], which has been demonstrated to be involved in the insulin-induced enhancer effect on* Slc2a4* transcription [40]. Thus, miR-21a-5p by decreasing PTEN increases PI3K/AKT activity, which would enhance* Slc2a4*/GLUT4 expression. Unfortunately, the study by Ling and colleagues [117] did not evaluate* Slc2a4* mRNA or total cellular GLUT4 protein expression to reinforce this hypothesis.

Another indirect regulation of GLUT4 is attributed to miR-29a-3p [118], which was seen to target the* Ppard* mRNA (peroxisome proliferator-activated receptor delta) suppressing PPARD protein, which in turn reduced the PGC1A (peroxisome proliferator-activated receptor gamma coactivator 1-alpha); the latter is an important enhancer of* Slc2a4* transcription [120]. In fact, overexpression of miR-29a-3p in skeletal muscle cell induced a GLUT4 reduction, bearing in mind that the miR-29a-3p can also play a direct effect on* Slc2a4* mRNA, as commented on above.

Predictable direct regulation of* Slc2a4*, indicated by in silico analysis and/or correlation between expression of GLUT4 and miRNA, have been proposed to miR-29c-3p, miR-29a-3p, and miR-222-3p [115, 118, 121].

Increased levels of miR-29c-3p with reduced GLUT4 content were observed in cardiomyocytes of obese animals, suggesting an inverse relationship between GLUT4 and miRNA-29c-3p; however, this hypothesis needs to be validated [121]. In addition, upregulation of miR-29a-3p was observed in skeletal muscle of rats with intrauterine growth restriction; and overexpression of miR-29a-3p in muscle C2C12 cells induced a reduction in* Slc2a4* mRNA and GLUT4 protein [118]. However, the direct effect of both miR-29c-3p and miR-29a-3p upon GLUT4 expression remains to be demonstrated. In silico analysis points out* Slc2a4* mRNA as target to miR-222-3p; however, whether the* Slc2a4*/GLUT4 regulation observed in response to miR-222-3p involves a direct effect of this microRNA upon* Slc2a4* mRNA, or it is just secondary to the ESR1 regulation [115], as commented on above, remains to be determined.

Finally, in obese dogs that have been subjected to weight loss, the expression of both miR-23a and miR-107 was downregulated, and that was accompanied by decreased* Slc2a4* mRNA expression; however, in this experimental condition* AKT2* and* RAC1* were also repressed, and thus the* Slc2a4* regulation might be direct and/or indirect [106].

**Figure 1:**
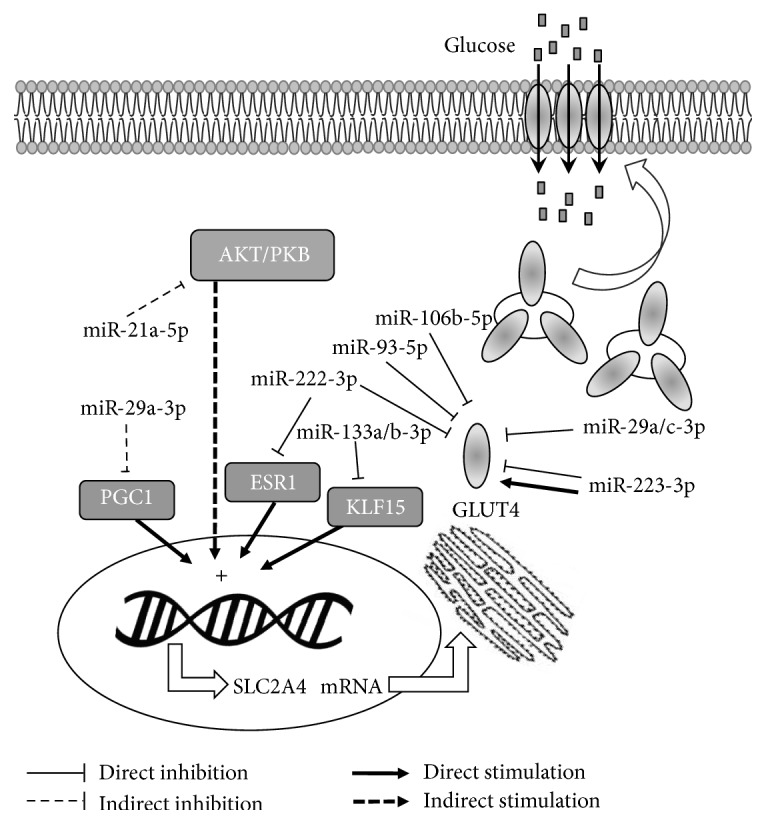
*SLC2A4*/GLUT4 expression regulation by miRNAs in muscle and adipose tissues. Regulation have been proposed to occur directly in GLUT4 translation and/or in transcription factors that regulate* SLC2A4* transcription, and, consequently, GLUT4 expression. Several mechanisms are involved in translocation of GLUT4 storage vesicles to the plasma membrane, increasing GLUT4 density and glucose transport. PI3K, phosphatidylinositol 3-kinase; AKT, RAC-alpha serine/threonine-protein kinase; PGC1A, peroxisome proliferator-activated receptor gamma coactivator 1-alpha; ESR1, estrogen receptor 1; KLF15, Krüeppel-like factor 15; GLUT4, solute carrier family 2, facilitated glucose transporter member 4;* SLC2A4*, solute carrier family 2 member 4 gene; miR, microRNA.

Table 2 shows the miRNAs that have been related to GLUT4 expression, and Figure 1 shows a schematic summary of direct and indirect regulation of GLUT4.

## 8. Conclusions

In adipose and muscle tissues, several miRNAs have already been described as repressors of GLUT4 expression and, consequently, of tissue glucose disposal. In addition, in skeletal muscle, some miRNAs have been linked to the machinery involved in GLUT4 translocation. Moreover, the expression of these miRNAs has been described as altered in humans or experimental models with insulin resistance, thus revealing their potential participation in the pathophysiology of diabetes. Among these miRNAs, miR-29a-3p, miR-29c-3p, miR-93-5p, miR-222-3p, miR-223-3p, and miR-106b-5p are proposed to target the* Slc2a4* 3′UTR, whereas miR-133a-3p, miR-133b-3p, and miR-222-3p are proposed to target enhancers of* Slc2a4* gene transcription. Besides, these and other miRNAs have been related to the expression/activity of proteins involved in the GLUT4 machinery translocation, which might also impact tissue glucose disposal and glycemic homeostasis as well. Thus, the study of miRNAs potentially involved in the regulation of GLUT4 expression and translocation represents an important field that can contribute to establishing preventive and/or therapeutic approaches for insulin resistance and diabetes.

## Figures and Tables

**Table 1:** Insulin resistance-induced regulation of miRNAs related to proteins of the GLUT4 translocation machinery in skeletal muscle.

miRNA^*∗*^	Condition	miRNA expression	Proposed target^*∗∗*^ and its regulation	Reference
let-7f-5p	T2DM human and mouse	↑	↓ *INSR*,* IRS2*	Zhu et al. [100]
miR-10b-5p	GK rat	↓	—	Herrera et al. [97]
miR-15b-5p	T2DM human	↓	↓ *INSR*,* IRS1*, ↑ *PIK3R1*	Bork-Jensen et al. [101]
miR-16-5p	Obese rat and mouse	↓	↓ *MTOR*, *RPS6KB1*	Lee et al. [108]
miR-23a	Loss of weight in obese dog	↓	↓ *RAC1, AKT2*	Uribe et al. [106]
miR-24-3p	GK rat	↓	↑ *MAPK14*	Huang et al. [99]
miR-29a/b/c-3p	GK rat, IR-obese mouse	↑	↓ *AKT2, IRS1*	He et al. [107]; Yang et al., [103]
miR-107	Loss of weight in obese dog	↓	↓ *RAC1, AKT2*	Uribe et al. [106]
miR-133a-3p	T2DM human	↓	—	Gallagher et al. [98]
miR-135a-5p	T2DM mouse, IR-C2C12 cell	↑	↓ *INSR, IRS2*	Agarwal et al. [104]; Honardoost et al. [102]
miR-144-3p	T2DM rat	↑	↓ *IRS1*	Karolina et al. [105]
miR-206	T2DM human	↓	—	Gallagher et al. [98]

^*∗*^The nomenclature of miRNAs is in accordance with the “miRBase Sequence Database-Release 21.” ^*∗∗*^Gene names are based on HGNC (HUGO Gene Nomenclature Committee), and despite some date being related to mouse/rat gene, description was related to human gene (capital letters). IR, insulin resistance/resistant; T2DM, type 2 diabetes mellitus; GK, Goto-Kakizaki; *INSR*, insulin receptor; *IRS1*, insulin receptor substrate 1; *IRS2*, insulin receptor substrate 2; *MAPK14*, mitogen-activated protein kinase 14; *AKT2*, AKT serine/threonine kinase 2; *PI3KR1*, phosphoinositide-3-kinase regulatory subunit 1; *MTOR*, mechanistic target of rapamycin; *RPS6KB1*, ribosomal protein S6 kinase B1; *RAC1*, ras-related C3 botulinum toxin substrate 1 (rho family, small GTP binding protein Rac1).

**Table 2:** miRNAs related to the *Slc2a4*/GLUT4 expression in insulin resistance.

miRNA^*∗*^	Species/tissue	Description	Reference
miR-23a	Dog/skeletal muscle	Downregulated after loss of weight, together with decreased *Slc2a4* mRNA	Uribe et al. [106]

miR-29c-3p	Mouse/heart	Upregulated in heart of obese mice, together with decreased GLUT4	Guedes et al. [121]

miR-29a-3p	Rat and mouse/ skeletal muscle	Upregulated in muscle of rats with intrauterine growth restriction; overexpression in C2C12 myocyte decreased GLUT4	Zhou et al. [118]

miR-93-5p	Human/adipose	Upregulated in adipose tissue of women with PCOS and/or IR; overexpression in adipocyte decreased GLUT4	Chen et al. [111]

miR-106b-5p	Rat/skeletal muscle	Upregulated in muscle of rats with T2DM; overexpression in L6 myocyte decreased GLUT4 and glucose uptake	Zhou et al. [114]

miR-107	Dog/skeletal muscle	Downregulated after loss of weight, together with decreased *Slc2a4* mRNA	Uribe et al. [106]

miR-133a/b-3p	Rat/heart	Overexpression in cardiomyocyte decreased GLUT4 and insulin-stimulated glucose uptake	Horie et al. [116]

miR-222-3p	Human/adipose	Upregulated in adipose tissue of women with GDM; overexpression in adipocyte decreased *Slc2a4 *mRNA	Shi et al. [115]

miR-223-3p	Human/adipose	Upregulated in adipose tissue of women with IR; cell overexpression decreased GLUT4 and glucose uptake	Chuang et al. [112]

miR-223-3p	Human and rat/heart	Upregulated in heart of humans with T2DM; overexpression in rat cardiomyocyte increased GLUT4	Lu et al. [113]

^*∗*^The nomenclature of miRNAs is in accordance with the “miRBase Sequence Database-Release 21.” IR, insulin resistance; GDM, gestational diabetes mellitus; PCOS, polycystic ovary syndrome; T2DM: type 2 diabetes mellitus.
